# Post-COVID-19 Patients Who Develop Lung Fibrotic-like Changes Have Lower Circulating Levels of IFN-β but Higher Levels of IL-1α and TGF-β

**DOI:** 10.3390/biomedicines9121931

**Published:** 2021-12-17

**Authors:** Chiara Colarusso, Angelantonio Maglio, Michela Terlizzi, Carolina Vitale, Antonio Molino, Aldo Pinto, Alessandro Vatrella, Rosalinda Sorrentino

**Affiliations:** 1 Department of Pharmacy (DIFARMA), University of Salerno, Via Giovanni Paolo II 132, 84084 Fisciano, Italy; ccolarusso@unisa.it (C.C.); mterlizzi@unisa.it (M.T.); pintoal@unisa.it (A.P.); 2 Department of Medicine and Surgery, University of Salerno, Baronissi Campus, Via S. Allende, 84081 Baronissi, Italy; amaglio@unisa.it (A.M.); carolinavitale.med@gmail.com (C.V.); avatrella@unisa.it (A.V.); 3 Department of Respiratory Medicine, Respiratory Division, University of Naples Federico II, Via S. Pansini 5, 80131 Naples, Italy; molinotonio@libero.it

**Keywords:** SARS-CoV-2, post-COVID-19, pulmonary fibrosis, inflammation, cytokines

## Abstract

Purpose: SARS-CoV-2 infection induces in some patients a condition called long-COVID-19, herein post-COVID-19 (PC), which persists for longer than the negative oral-pharyngeal swab. One of the complications of PC is pulmonary fibrosis. The purpose of this study was to identify blood biomarkers to predict PC patients undergoing pulmonary fibrosis. Patients and Methods: We analyzed blood samples of healthy, anti-SARS-CoV-2 vaccinated (VAX) subjects and PC patients who were stratified according to the severity of the disease and chest computed tomography (CT) scan data. Results: The inflammatory C reactive protein (CRP), complement complex C5b-9, LDH, but not IL-6, were higher in PC patients, independent of the severity of the disease and lung fibrotic areas. Interestingly, PC patients with ground-glass opacities (as revealed by chest CT scan) were characterized by higher plasma levels of IL-1α, CXCL-10, TGF-β, but not of IFN-β, compared to healthy and VAX subjects. In particular, 19 out of 23 (82.6%) severe PC and 8 out of 29 (27.6%) moderate PC patients presented signs of lung fibrosis, associated to lower levels of IFN-β, but higher IL-1α and TGF-β. Conclusions: We found that higher IL-1α and TGF-β and lower plasma levels of IFN-β could predict an increased relative risk (RR = 2.8) of lung fibrosis-like changes in PC patients.

## 1. Introduction

Severe acute respiratory syndrome coronavirus-2 (SARS-CoV-2) has caused a world-wide pandemic inducing a disease known as coronavirus disease 19 (COVID-19) [1]. The dissemination of SARS-CoV-2 has changed our lives at different levels such as lifestyle, social relationships, and health approaches. 

COVID-19 is a life-threatening disease leading to bilateral pneumonia and respiratory failure. The underlying mechanism/s why the majority of infected patients present mild or moderate home-cured symptoms compared to the lower percentage of people who develop a severe disease is still elusive. It is now well-known that a “cytokine storm” is responsible of SARS-CoV-2 severity [2] in that COVID-19 infected patients need hospitalization for oxygen supplementation and, in some cases, undergo intensive care. In particular, it was found that deficiencies in type I interferons (IFN-I), either induced by inherited mutations [3] or due to the development of antibodies that ‘neutralize’ IFN-I [4], may underlie severe grade of COVID-19.

Another important issue correlated to COVID-19 is that a significant number of patients, although negative to the oral-pharyngeal swab, experience prolonged symptoms, herein called post COVID-19 (PC) [5]. Davis and collaborators found that a cohort of PC patients presented prolonged symptoms ranging from neuropsychiatric, reproductive, cardiovascular, musculoskeletal, immunological, head-ear-eye-nose-throat, pulmonary, gastrointestinal, and dermatologic symptoms up to multiorgan systemic dysfunction, impacting morbidity, mortality, and quality of life [6].

Pulmonary fibrosis can occur after viral pneumonia such as that induced by SARS-CoV and Middle East respiratory syndrome coronavirus (MERS) infection, which, in some cases, leads to pulmonary dysfunction for a period longer than two years [1,2]. Similarly, SARS-CoV-2 can cause lung fibrotic dysfunction/s post infection. However, the mechanism/s underlying SARS-CoV-2-derived pulmonary fibrosis are still not well defined. The aim of this study was to understand whether blood biomarkers such as pro-inflammatory markers (i.e., C reactive protein, CRP) and cytokines (i.e., IFN-I) could predict lung fibrosis-like changes in PC patients.

In the attempt to prevent and early therapeutically treat PC patients showing pulmonary fibrosis-like symptoms, we tried to understand what circulating biomarkers were involved. The results from this study could be of clinical help to immediately characterize and discriminate pulmonary fibrosis susceptible PC patients

## 2. Materials and Methods

### 2.1. Human Samples

Blood samples were collected by healthy volunteers, vaccinated (VAX) subjects, and post-COVID-19 (PC) patients recruited at the “Monaldi-Azienda Ospedaliera (AORN)-Ospedale dei Colli” Hospital in Naples, Italy. All subjects and patients signed an informed consent before using blood samples as required by the Ethical Committee Board of the hospital, which approved the experimental protocols in accordance with the guidelines and regulations provided by the Review Board (protocol n.422/2017, 729/2020). Blood from healthy subjects was collected according to negative swab to SARS-CoV-2. Vaccinated subjects underwent mRNA vaccination and were negative to SARS-CoV2 infection.

The age of post-COVID-19 patients (PC; n = 52) and healthy subjects (Healthy; n = 17) had a mean of 50 ± 10 years, instead, the mean age of anti-SARS-CoV-2 VAX subjects (n = 27) was 30 ± 10 years. All subjects/patients had no previous history of allergic diseases or chronic respiratory conditions. Blood was collected by PC patients for 1–3 months by negative oral-pharyngeal swab to detect SARS-CoV-2 infection. PC patients were classified as moderate COVID patients (n = 29) who did not need hospitalization and were cured at home, or severe COVID patients (n = 23) who requested hospitalization and oxygen therapy according to the clinical need. In addition, within the cohort of PC patients, the presence of fibrosis-like changes was assessed according to functional and clinical parameters such as spirometry, FEV1, FVC, and the presence of ground-glass opacities and reticular/fibrotic areas at the chest CT scan, as described below. Clinical and therapeutical features of PC patients are reported in Table 1.

Blood was collected and used within 24 h, centrifuged at 3750 rpm for 10 min to spin down all blood cells to collect plasma.

### 2.2. Cytokine and Chemokine Measurements

IL-1α, IL-2, IL-6, IFN-β, IL-18, IL-33, TGF-β, CXCL8, and CXCL10 levels were measured in cell-free plasma using commercially available enzyme-linked immunosorbent assay kits (ELISAs) (Diaclone, Buckingham, UK; R&D, Abingdon, UK).

### 2.3. C reactive Protein (CRP) and Complement Complex C5b-9 Measurements

Plasma levels of CRP and complement complex C5b-9 were measured according to the manufacturer’s instructions (Elabscience, Houston, TX, USA).

### 2.4. LDH Levels

The levels of lactate dehydrogenase (LDH) were measured by using a commercially available kit (Sigma, Rome, Italy) following the manufacturer’s instructions.

### 2.5. Chest CT Scans

Chest CT scans were performed 1–3 months after the first negative oral-pharyngeal swab. Patients were examined in the supine position, covering the area from the apex of the lung to the costophrenic angle with a scanning layer thickness and layer spacing of 0.5–2 mm (HRTC). The pulmonary involvement was measured by expert radiologists by applying a semi-quantitative scoring system based on the pulmonary area involved with fibrosis [7]. Each of the five lung lobes was visually scored from 0 to 5 as: 0, no involvement; 1, <5% involvement; 2, 25% involvement; 3, 26–49% involvement; 4, 50–75% involvement; 5, >75% involvement. The total CT score was the sum of the individual lobar scores and ranged from 0 (no involvement) to 25 (maximum involvement) [8]. In this study, we evaluated the presence of fibrotic or non-fibrotic patterns by applying a qualitative measure closely associated with the semi-quantitative score previously described. Presence of fibrotic pattern was considered in patients with a score >5.

### 2.6. Statistical Analysis

Data are reported as median. Statistical differences were assessed with Mann–Whitney U test, two-sided Fisher’s exact test, and Chi-square test where needed. *p* values less than 0.05 were considered as significant. The statistical analysis was performed by using GraphPad Prism version 9.0.0 (San Diego, CA, USA).

## 3. Results

### 3.1. Circulating Levels of Inflammatory Markers Are Still Present in Post-COVID-19 Patients

**Figure 1:**
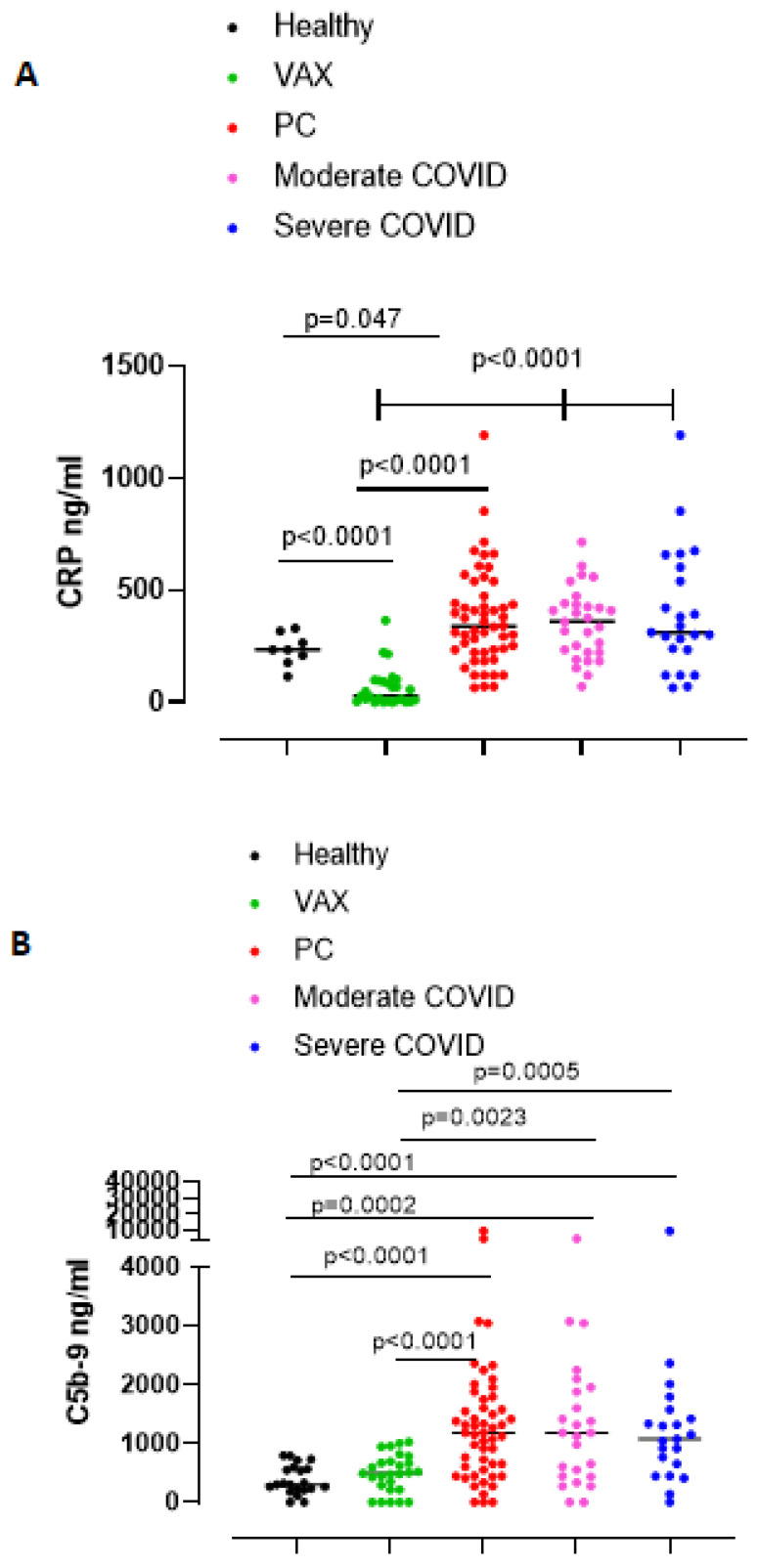
(**A**) Plasma levels of C-reactive protein (CRP) and (**B**) complement complex C5b-9 were significantly higher in PC patients than healthy (black dots) and vaccinated (VAX, green dots) subjects. Post-COVID-19 (PC) patients (red dots) were further stratified as moderate or severe patients (pink and blue dots, respectively). Data are expressed as median. Statistical analysis was performed according to the Mann–Whitney U test.

SARS-CoV-2 infected patients develop a systemic inflammatory syndrome [9]. C reactive protein (CRP) was considered as a systemic inflammatory marker associated with the severity of the infection [10]. We found that plasma CRP was statistically (*p* = 0.047) higher in PC patients than in healthy subjects (Figure 1A, red vs. black dots). In sharp contrast, anti-SARS-CoV-2 vaccinated (VAX) subjects had significantly lower levels of CRP than healthy subjects and PC patients (Figure 1A, green dots vs black or red dots). To note, VAX subjects had even lower levels of CRP than healthy subjects, implying that the vaccination does not induce the systemic inflammatory syndrome such as during SARS-CoV-2 infection [10]. To understand whether there were differences in plasma CRP levels underlying the grade of COVID-19, we stratified PC patients as moderate or severe according to the hospitalization where they needed oxygen therapy. Moderate (Figure 1A, pink dots) and severe (Figure 1A, blue dots) PC patients had similar CRP levels post infection. Furthermore, the levels of lactate dehydrogenase (LDH), another circulating inflammatory marker, were significantly higher in both moderate and severe PC patients compared to healthy subjects (Supplementary Figure S1 in the Supplementary Materials).

COVID-19 was also described as affected by alteration/s of the coagulation pathway as well as of the complement cascade [1]. Similarly, we measured the activation of the complement C3 and C5 by detecting the C3a and C5b-9 complex, respectively. We did not observe any difference for circulating C3a in the three groups (data not shown). Instead, the complement complex C5b-9 was significantly higher in the blood of PC patients than healthy and VAX subjects (Figure 1B, red vs. black and green dots). However, we did not find differences in the levels of C5b-9 between moderate vs. severe PC patients (Figure 1B, pink vs. blue dots).

**Figure 2:**
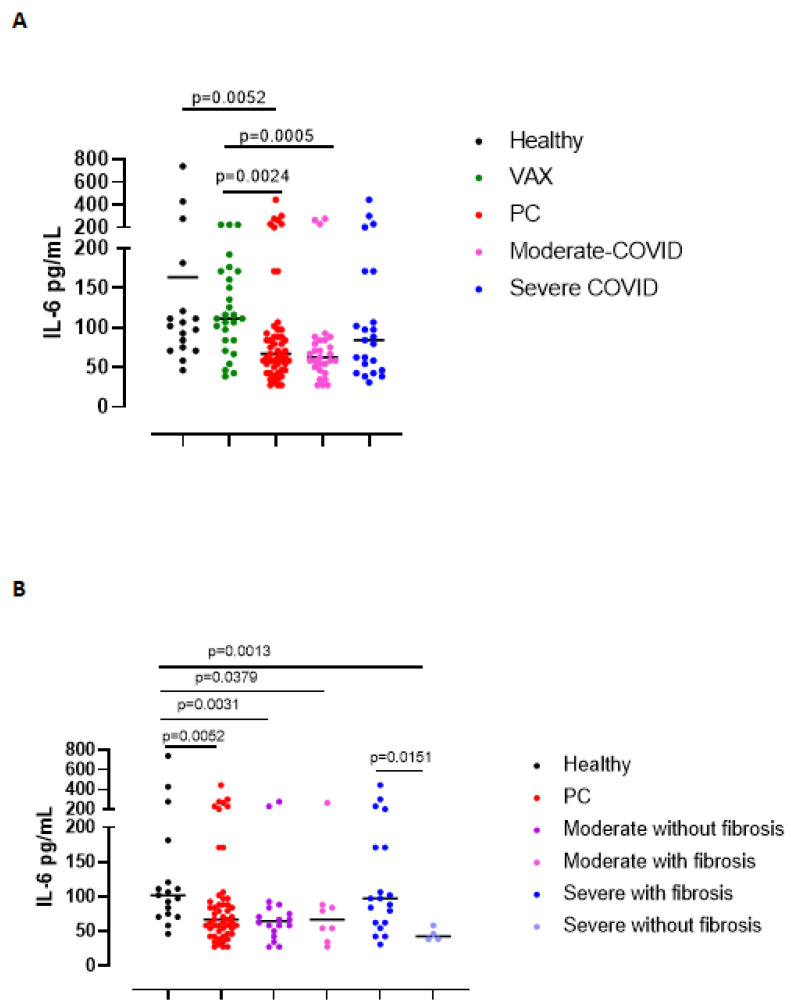
Circulating levels of IL-6 in moderate and severe PC patients (**A**), as well as in moderate and severe PC patients with or without fibrosis-like symptoms (**B**) were reduced compared to healthy (black dots) and vaccinated (VAX, green dots) subjects. (**A**) Plasma levels of IL-6 were measured according to the grade of the disease (moderate or severe, pink and blue dots, respectively) and (**B**) to the establishment of lung fibrosis-like symptoms (moderate or severe PC patients with or without fibrosis, pink or blue dots and purple and bright blue dots, respectively). Data are expressed as median. Statistical analysis was performed according to the Mann–Whitney U test.

Based on the fact that IL-6 is one of the cytokines observed during the “cytokine storm” typical of COVID-19 [11], we measured plasma levels of IL-6. Circulating levels of IL-6 were significantly reduced in all PC blood samples compared to healthy and VAX subjects (Figure 2A). No differences in circulating IL-6 levels were observed in PC patients stratified as moderate vs severe (Figure 2A), or as with or without fibrosis (Figure 2B, pink vs blue dots).

These data imply that PC patients, although the negative oro-pharyngeal swab, presented circulating inflammatory markers such as the CRP and C5b-9 complex, but not IL-6.

### 3.2. Circulating Levels of IFN-β Are Still High in Post-COVID-19 Patients Who Did Not Have Lung Fibrosis-like Changes Post Infection

**Figure 3:**
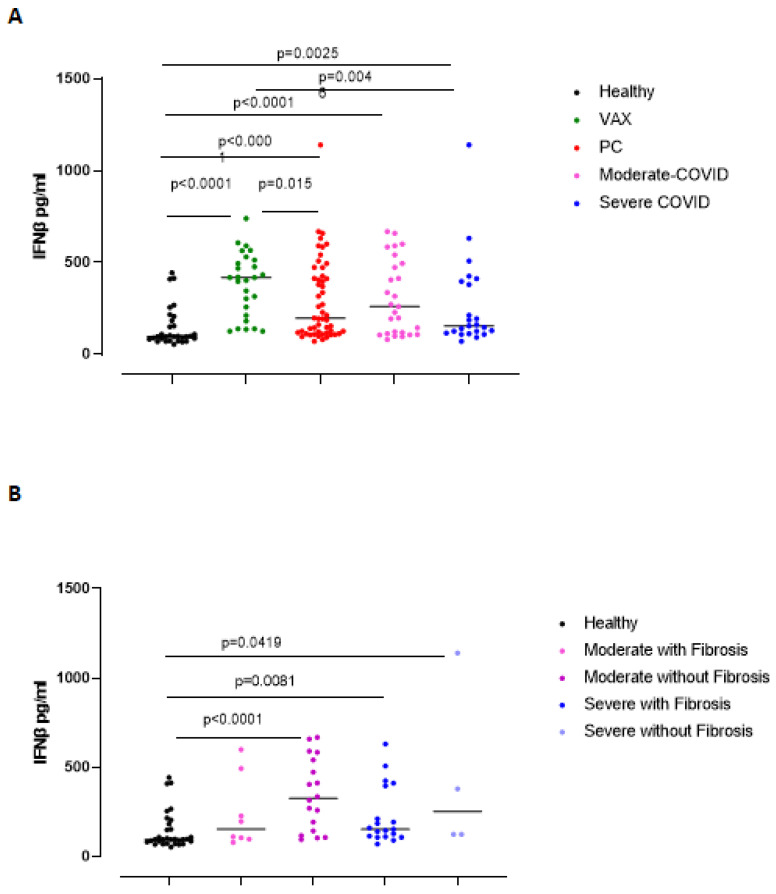
Plasma levels of IFN-β were increased in PC patients but reduced according to the presence of symptoms and signs of lung fibrosis. (**A**) Circulating levels of IFN-β were higher in vaccinated (VAX) subjects than in healthy and PC patients (green vs. black and red dots); these levels did not discriminate moderate (pink dots) vs. severe (blue dots) PC patients. Instead, (**B**) the absence of fibrosis in PC patients was characterized by higher levels of IFN-β in moderate (purple dots) and severe (bright blue dots) PC patients. Data are expressed as median. Statistical analysis was performed according to the Mann–Whitney U test.

Type I interferons are anti-viral cytokines that were described in response to SARS-CoV-2 infection [12]. We found that PC patients still presented higher levels of circulating IFN-β compared to healthy subjects (Figure 3A, red vs. black dots). It is interesting to note that VAX subjects had higher levels of IFN-β compared to PC patients and healthy subjects (Figure 3A, green vs. red and black dots). Patients who had moderate grade of COVID-19 showed a tendency to have higher levels of circulating IFN-β than those who had severe COVID-19 (media ± SEM: 308.2 ± 39 vs. 264 ± 51.1 pg/mL), although no statistical difference was observed between the two groups (Figure 3A, pink vs. blue dots). In addition, PC patients who did not develop pulmonary fibrosis-like changes (moderate without fibrosis, purple dots, and severe without fibrosis, light blue dots) had higher levels of IFN-β than those who had fibrosis-like changes (moderate with fibrosis, pink dots, and severe with fibrosis, blue dots) (Figure 3B).

**Figure 4:**
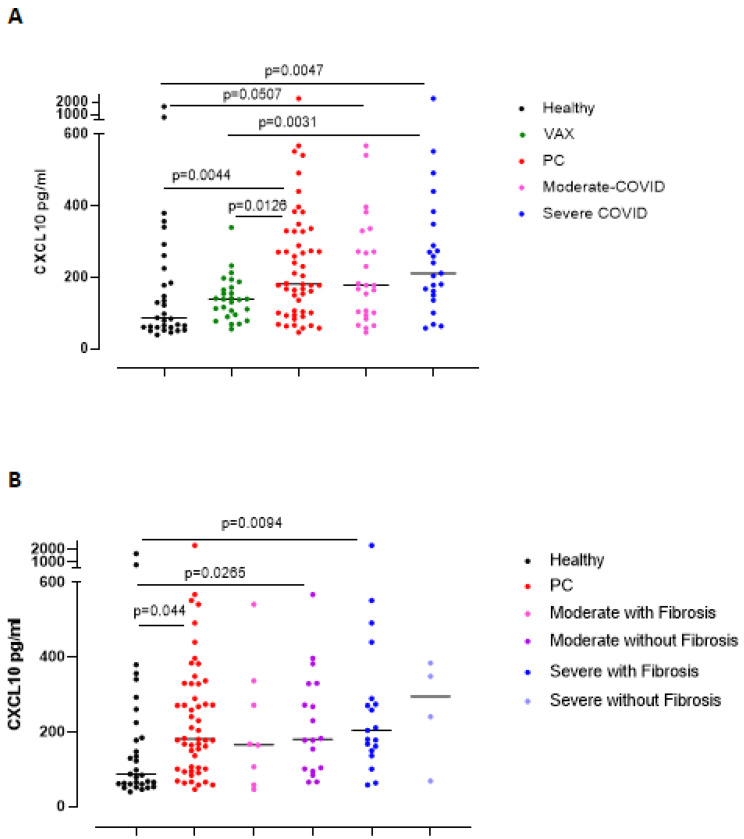
Plasma levels of CXCL10 were increased in PC patients according to the severity of the disease and to the presence of symptoms and signs of lung fibrosis. (**A**) Circulating CXCL10 levels were higher in severe PC patients who developed symptoms and signs of lung fibrosis compared to heathy and VAX subjects (blue dots vs. black and green dots). (**B**) CXCL10 levels were evaluated according to lung fibrosis-like symptoms. Data are expressed as median. Statistical analysis was performed according to the Mann–Whitney U test.

To confirm the relevance of IFN-β, we analyzed an associated chemokine, CXCL10 [13]. The levels of CXCL10 were significantly higher in PC patients than in the healthy and VAX subjects (Figure 4A, red vs. black and green dots). Severe grade of COVID-19, differently than what observed for IFN-β, induced higher levels of CXCL10 compared to healthy and VAX subjects (Figure 4A, blue vs. black and green dots), implying the prominent role of an active IFN-dependent immunity against the virus. Indeed, severe and moderate PC patients with lung fibrosis-like changes had higher levels of CXCL10 than healthy subjects (Figure 4B, pink and blue dots).

**Figure 5:**
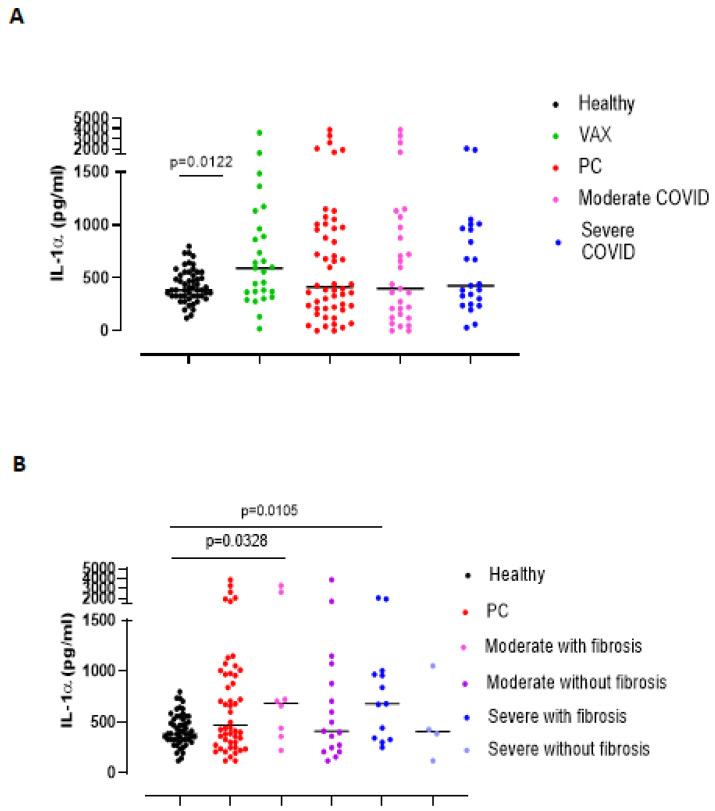
Plasma levels of IL-1α were increased in PC patients who developed symptoms and signs of pulmonary fibrosis, independently of the grade disease. (**A**) Circulating levels of IL-1α were not altered in the healthy and PC patients. (**B**) IL-1α levels were evaluated according to the establishment of lung fibrosis symptoms (moderate or severe PC patients with or without fibrosis, pink or blue dots and purple and bright blue dots, respectively). Data are expressed as median. Statistical analysis was performed according to Mann Whitney U test.

Another cytokine involved in lung fibrosis is IL-1α [14,15,16]. We found that plasma levels of IL-1α in all PC patients were not statistically different than in healthy subjects (Figure 5A, red vs. black dots), but VAX subjects had higher levels of IL-1α (Figure 5A, green dots), likely due to the activation of the immune system after the vaccine boost. Similarly, the stratification of PC patients as moderate vs severe did not show any difference in IL-1α levels (Figure 5A, pink and blue dots). In contrast, when PC patients were stratified as with or without fibrosis-like symptoms, we observed that IL-1α was higher in both moderate and severe PC patients with fibrosis-like changes than healthy subjects (Figure 5B, pink and blue vs. black dots), although no statistical differences were registered.

In addition, we did not find increased levels of IL-2, IL-18, IL-33, and CXCL8 in PC patients compared to healthy subjects (Supplementary Figure S2 in the Supplementary Materials). It has to be noted that VAX subjects had significantly higher levels of IL-18 than PC patients (Supplementary Figure S2B, green vs. red dots, in the Supplementary Materials), likely due to the effectiveness of the vaccine.

### 3.3. The Immunosuppressive/Pro-Fibrotic TGF-β Is Higher in Severe Post-COVID-19 Patients Who Had Pulmonary Fibrotic-like Events

**Figure 6:**
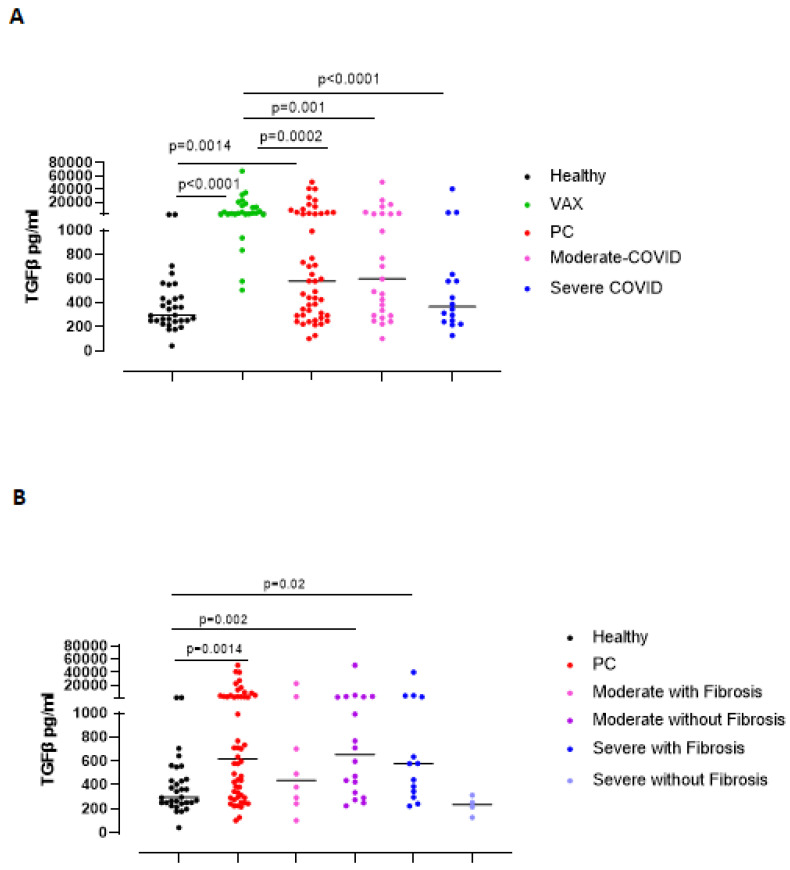
Circulating TGF-β levels were higher in severe post-COVID-19 patients with lung fibrosis events. (**A**) Plasma levels of TGF-β were measured in healthy, vaccinated (VAX) subjects (black and green dots, respectively), and post-COVID-19 (PC) patients (red dots), according to the grade of the pathology (moderate vs. severe, pink and blue dots, respectively). (**B**) TGF-β levels were evaluated according to the establishment of lung fibrosis symptoms (moderate or severe PC patients with or without fibrosis, pink or blue dots and purple and bright blue dots, respectively). Data are expressed as median. Statistical analysis was performed according to the Mann–Whitney U test.

TGF-β has long been proposed as a key molecule in the pathogenesis of lung fibrosis [14,17]. In this regard, we found that PC patients had higher levels of TGF-β compared to healthy subjects (Figure 6A, red vs. black dots). VAX subjects had very high levels of this cytokine (Figure 6A, green dots), most likely due to the immunosuppressive arm that establishes post vaccine injection [18]. No statistical differences were observed between moderate vs severe PC patients (Figure 6A, pink vs. blue dots). Stratifying PC patients according to lung fibrosis-like changes, we found that moderate PC patients without fibrosis had higher levels of TGF-β than healthy subjects (Figure 6B, purple vs. black dots); however, moderate PC patients with fibrosis had a slight increase in TGF-β compared to healthy subjects (Figure 6B, pink vs. black dots). Instead, severe PC patients who presented signs of lung fibrosis had higher levels of TGF-β than both healthy subjects (Figure 6B, blue vs black dots) and severe PC patients without fibrosis (Figure 6B, blue vs. bright blue dots).

### 3.4. Severe COVID-19 Patients Display Higher Susceptibility to Lung Fibrosis

**Figure 7:**
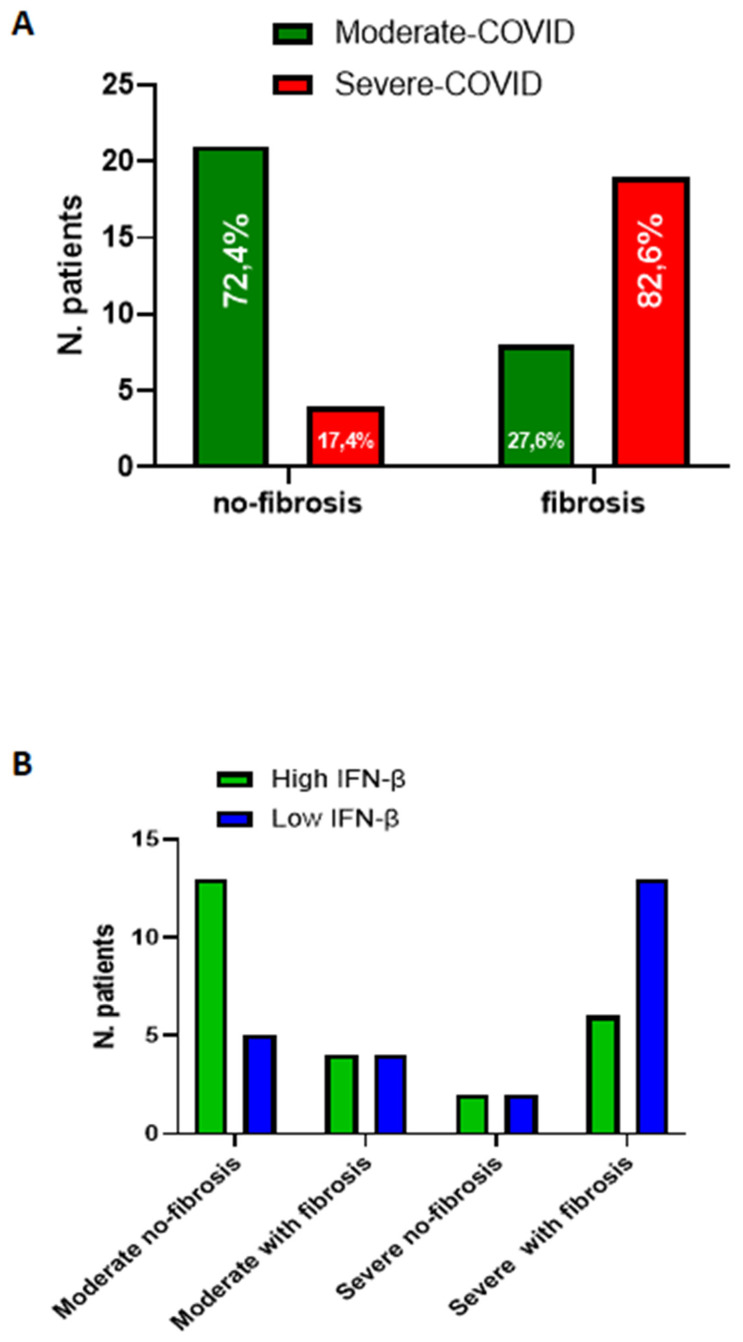
Discrimination among PC patients with or without fibrosis-like symptoms according to plasma levels of IFN-β. (**A**) The contingency graph that describes that lung fibrosis-like changes are more frequent in severe (red bar, 82.6%) than moderate (green bar, 72.4%) COVID-19 patients. Fisher’s exact test was used to determine the possible association between COVID-19 and lung fibrosis events (*p* < 0.0001). (**B**) Fraction of total was analyzed according to the levels of IFN-β based on a cut-off (194.3 pg/mL) obtained by receiver operating curve (ROC) analysis; in this latter analysis, the Chi-square test for trend (*p* = 0.0154) was applied.

To understand whether COVID-19 induced lung fibrosis, we analyzed the group of patients according to the grade of the disease. We found that patients who had severe infection (n = 23/52, 44.2% of the total) presented signs of lung fibrosis post infection (n = 19/23, 82.6%) (Figure 7A, red bar in fibrosis section). This phenomenon was statistically significant according to Fisher’s exact test (*p* < 0.0001). Instead, patients who had a moderate grade of COVID-19 (n = 29/52, 55.8% of the total) did not show lung fibrosis signs (n = 21/29, 72.4%) (Figure 7A, green bar in no-fibrosis section). Importantly, among moderate COVID-19 patients, 8 out of 29 (27.6%) patients presented symptoms and fibrotic areas at the chest CT scan (Figure 7A, green bar in the fibrosis section); 4 patients out of 23 (17.4%), who presented a severe grade of COVID-19, did not develop lung fibrosis (Figure 7A, red bar in no fibrosis section). Of note, this latter group of patients, although the number was limited (n = 4), had lower levels of the pro-fibrotic IL-1α (Figure 5B) and TGF-β (Figure 6B), but higher levels of IFN-β (Figure 3B). Considering IFN-β as the discriminating cytokine between fibrotic vs non-fibrotic PC patients in Table 2, we performed a Chi-square test taking into consideration a receiver operating curve (ROC) analysis to highlight a cut-off value of the cytokine (194.3 pg/mL). We found that severe PC patients who presented lung fibrosis-like changes had lower levels of IFN-β (Figure 7B, blue bar on the right, as indicated by the arrow). Instead, patients with moderate COVID-19 who presented higher levels of IFN-β had no signs of lung fibrosis-like changes (Figure 7B, green bar on the left, as indicated by the arrow). Interestingly, the group of patients defined as moderate with fibrosis (n = 8 out of 29) and severe without fibrosis (n = 4 out of 23) presented fibrosis-like symptoms or not, independently of IFN-β levels, implying other yet unidentified blood biomarker/s for PC patients.

## 4. Discussion

Several reports have described clinical and epidemiological features of patients affected by COVID-19. The common symptoms of SARS-CoV-2 infection are fever, cough, myalgia and/or fatigue, dyspnea, gastrointestinal dysfunctions, and prominent respiratory tract impairment [1]. All hospitalized patients show abnormalities in chest CT images, characterized by grinding glass-like and consolidation areas in 98% of the cases reporting bilateral lung impairment at the basis of bilateral interstitial pneumonia [1]. The morbidity is mainly due to respiratory failure typical of acute respiratory distress syndrome (ARDS), but the mortality underlies multiple organ failure [1]. Hematopoietic alterations are characterized by lymphopenia, thrombocytopenia, thromboembolism, and increased plasma levels of inflammatory cytokines and chemokines (i.e., IL-6, CCL2, TNF-α) [18].

Besides the symptoms during SARS-CoV-2 infection, another important issue that rises post infection is related to long COVID-19 syndrome, herein PC. Some PC patients develop lung fibrosis-like changes, which especially occur in some patients who need hospitalization [19]. In our cohort of PC patients, we found that 27 out of 52 patients (52%) presented ground-glass opacities and reticular/fibrotic areas at the chest CT scan. To identify circulating biomarkers able to discriminate PC patients undergoing lung fibrosis-like changes, we measured different well-known inflammatory mediators. We found that CRP, complement complex C5b-9, and LDH were still higher in PC patients, despite the infection being abolished and despite the severity of the pathology. Instead, IL-6, a cytokine that was described as involved in the typical COVID-19-related “cytokine storm” [11], was lower in all PC patients who had similar levels as the healthy subjects. This could imply the rescue from infection.

Of note, subjects who received the anti-SARS-CoV-2 vaccine (mRNA-based vaccine) did not show any alteration of CRP, C5b-9 complex, and IL-6, implying that the vaccine solely boosts the immune system as, instead, confirmed by higher levels of IFN-β, IL-1α, IL-18, and TGF-β. The presence of these cytokines leads to suppose that CD4+ Th1 and CD8+ T cytotoxic cells together with the memory immunity were increased in vaccinated subjects, implying the establishment of a specific immunity to combat SARS-CoV-2 infection [18,20,21].

PC patients with lung fibrosis-like symptoms had higher levels of IL-1α and TGF-β, but lower levels of IFN-β, unlike the vaccinated (VAX) subjects and PC patients who did not show fibrosis-like signs. Therefore, the inverse relationship between IFN-β vs IL-1α and TGF-β could be at the crossroad, defining PC patients susceptible to lung fibrosis. In our previous study, we observed that IL-1α and TGF-β were highly released by peripheral blood mononuclear cells (PBMCs) obtained by patients with idiopathic pulmonary fibrosis (IPF) [14]. Here, we found that IL-1α and TGF-β were higher in patients with signs of lung fibrosis compared to IFN-β in PC patients. IFN-β is a well-known antiviral cytokine [12], which relevance during SARS-CoV-2 infection has been widely described. In particular, type I interferons have been related to the grade of the disease [3,4], in that the absence of this cytokine could be at the basis of a severe grade of COVID-19. Zhang and colleagues found that mutations of type I interferon genes are responsible for COVID-19 severity [3]. On the other hand, the neutralization of IFN type I due to endogenous antibodies favored the severity of COVID-19 [4]. In supporting, in our study, we found that IFN-β was significantly lower in severe PC compared to moderate PC patients. Fisher’s exact test performed on the levels of IFN-β showed that the relative risk (RR) of developing lung fibrosis-like changes was lower for PC who had higher levels of IFN-β compared to those who had lower levels (Figure 7B). VAX subjects had higher levels of IFN type I than the healthy, supporting both the efficacy/effectiveness of the vaccine (mRNA-based) to induce an anti-viral immunity, and the role of this cytokine to boost the immune system against SARS-CoV-2-derived spike protein.

On the other hand, severe or moderate PC patients who developed lung fibrosis-like changes had lower levels of IFN-β, but higher levels of IL-1α and TGF-β. TGF-β has long been proposed as a key molecule in the pathogenesis of lung fibrosis [14,17], playing a pivotal role in that it stimulates intrapulmonary fibroblasts to express high levels of collagen genes and mesenchymal cell-related markers such as α-smooth muscle actin (α-SMA) and vimentin [22]. IL-1α has been repeatedly reported as involved in chronic lung diseases [14,15,16]. In particular, IL-1α is involved in the phenotypic switch of lung fibroblasts to their inflammatory state during epithelial damage, herein suggested as an undesirable and harmful factor in fibrotic lung diseases [14,15,16]. Based on our previous studies [14], we found that higher levels of IL-1α and TGF-β, but lower levels of IFN-β, could reflect the increased RR (3-fold; RR = 2.8) of lung fibrosis-like changes in both severe and moderate COVID-19-affected patients post infection.

## 5. Conclusions

In conclusion, in the attempt to reveal blood biomarkers to predict PC patients undergoing pulmonary fibrosis-like changes, we believe that IL-1α and TGF-β compared to IFN-β could be of great relevance to avoid the molecular pro-fibrotic pattern typical of lung fibrosis, avoiding morbidity and mortality rates typical of lung fibrotic patients.

## Figures and Tables

**Table 1:** Characteristic of post-COVID-19 (PC) patients.

Patients	Sex	Grade	Fibrosis-Like Changes	Comorbidities
#1	F	Moderate	No	Hypertensive heart disease, type II diabetes mellitus, lymphatic stasis, cholangitis
#2	M	Severe	Yes	None
#3	F	Severe	Yes	Systemic arterial hypertension
#4	M	Moderate	Yes	Systemic arterial hypertension, COPD, type II diabetes mellitus
#5	M	Moderate	No	Intermittent bronchial asthma (treated with ICS/LABA as needed), obesity
#6	M	Severe	Yes	Systemic arterial hypertension, dyslipidemia
#7	F	Moderate	No	None
#8	F	Moderate	Yes	Recurrent bronchitis, systemic arterial hypertension
#9	F	Moderate	No	Diabetes mellitus
#10	M	Moderate	No	Hypercholesterolemia, previous pneumonia (2018)
#11	F	Severe	Yes	Systemic arterial hypertension, type 2 diabetes mellitus, hypercholesterolemia
#12	M	Moderate	No	Systemic arterial hypertension
#13	M	Moderate	Yes	Esophagogastroduodenitis, prostatitis
#14	M	Moderate	No	None
#15	M	Severe	Yes	Systemic arterial hypertension, ischemic heart disease, chronic renal failure, ischemic stroke
#16	F	Severe	No	Allergic bronchial asthma
#17	M	Severe	Yes	Systemic arterial hypertension
#18	M	Severe	No	None
#19	F	Moderate	No	Hashimoto’s thyroiditis
#20	M	Severe	Yes	Thrombophlebitis of the right lower limb
#21	M	Moderate	Yes	Chronic HBV-related liver disease, diabetes mellitus, systemic arterial hypertension, previous surgery for kidney stones
#22	F	Moderate	Yes	Systemic arterial hypertension
#23	F	Moderate	No	Bilateral hearing loss
#24	F	Severe	No	Hypertensive heart disease, dysthyroidism, hypercholesterolemia, atopy and allergic rhinosinusitis
#25	M	Severe	No	Gilbert’s syndrome, obesity
#26	M	Moderate	No	Dilated cardiomyopathy
#27	M	Moderate	No	None
#28	F	Moderate	No	Gastroesophageal reflux, colon dyskinesia
#29	M	Moderate	No	Hashimoto’s thyroiditis
#30	F	Severe	Yes	Bronchial asthma, allergic rhinitis
#31	M	Moderate	No	Hypercholesterolemia
#32	F	Moderate	Yes	None
#33	M	Moderate	No	None
#34		Moderate	No	None
#35	F	Severe	Yes	Depressive syndrome
#36	F	Severe	Yes	Hypothyroidism
#37	M	Severe	Yes	Gastroesophageal reflux
#38	F	Moderate	Yes	None
#39	M	Moderate	No	None
#40	M	Severe	Yes	Hypertension
#41	M	Moderate	No	Chronic ischemic heart disease, chronic atrial fibrillation, type 2 diabetes mellitus, hypertension, dyslipidemia
#42	M	Severe	Yes	None
#43	F	Severe	Yes	None
#44	M	Severe	Yes	Hypertension, gastroesophageal reflux disease, hemorrhoids
#45	M	Severe	Yes	Hypertension, rheumatoid arthritis
#46	M	Severe	Yes	Hypertension, type 2 diabetes mellitus
#47	M	Severe	Yes	Hypertension, gastroesophageal reflux disease
#48	F	Moderate	No	Sideropriva anemia-dystyroidism
#49	M	Moderate	Yes	None
#50	M	Moderate	No	Pulmonary emphysema
#51	M	Moderate	No	None
#52	M	Severe	Yes	None

**Table 2:** Analysis of cytokines in the group of post-COVID-19 (PC) patients according to lung fibrosis-like changes. Data are expressed as mean ± SEM (pg/mL).

Markers	Healthy	VAX	PC NO-Fibrosis Like Changes	PC Fibrosis Like Changes
CRP	236.2 ± 25.11	66.2 ± 16.4	Moderate: 360.9 ± 33.5 Severe: 376.5 ± 122.2	Moderate: 302.8 ± 51 Severe: 405.3 ± 65.91
C5b-9	382.5 ± 54.08	489.1 ± 62.38	Moderate: 158.5 ± 279.8 Severe: 126.7 ± 274.6	Moderate: 1058 ± 274.6 Severe: 1528 ± 488.1
LDH	183.9 ± 14.25	923.1 ± 150.5	Moderate: 10904 ± 1171 Severe: 9370 ± 3114	Moderate: 13565 ± 1768 Severe: 11496 ± 774.3
IL-6	163.4 ± 42.71	122.7 ± 10.74	Moderate: 81.89 ± 15.56 Severe: 45.48 ± 4.7	Moderate: 86.11 ± 26.9 Severe: 130.2 ± 24.02
IFN-β	145.4 ± 19.3	381.8 ± 33.2	Moderate: 349.1 ± 47.44 Severe: 442.6 ± 2.40	Moderate: 239.3 ± 70.3 Severe: 226.4 ± 37.4
CXCL-10	201.8 ± 55.4	141.4 ± 11.7	Moderate: 221.1 ± 32.65 Severe: 260.3 ± 71	Moderate: 211.4 ± 32.65 Severe: 339.9 ± 111.8
IL-1α	425.4 ± 21.1	764.3 ± 135.2	Moderate: 630 ± 210.9 Severe: 481.8 ± 207.3	Moderate: 1054 ± 431.4 Severe: 808.5 ± 168.7
TGF-β	393.8 ± 45.9	105.28 ± 298.7	Moderate: 4260 ± 3122 Severe: 227.3 ± 39.1	Moderate: 3606 ± 3238 Severe: 4358 ± 3283

Notes: CRP: C-reactive protein; C5b-9: complement complex C5b-9, LDH: lactate dehydrogenate; PC: post-COVID-19.

## Data Availability

The raw data supporting the conclusions of this article will be made available by the authors, without undue reservation.

## Supplementary Materials

The following are available online at https://www.mdpi.com/article/10.3390/biomedicines9121931/s1, Figure S1: Lactate dehydrogenate (LDH) levels were increased in post COVID-19 (PC) patients. Plasma levels of LDH were measured in healthy and vaccinated (VAX) subjects (black and green dots, respectively) and PC patients (red dots). LDH values were evaluated according to the grade of disease (moderate or severe, pink and blue dots, respectively). Data are expressed as median. Statistical analysis was performed according to the Mann–Whitney U test. Figure S2: Circulating levels of IL-2, IL-18, IL-33, and CXCL8 in PC patients and in healthy and vaccinated subjects. Plasma levels of IL-2 (A), IL-18 (B), IL-33 (C), and CXCL8 (D) were measured in healthy and vaccinated (VAX) subjects (black and green dots, respectively) and post COVID-19 (PC) patients (red dots). Data are expressed as median. Statistical analysis was performed according to the Mann–Whitney U test.

## References

[1] Colarusso C., Terlizzi M., Pinto A., Sorrentino R. (2020). A lesson from a saboteur: High-MW kininogen impact in coronavirus-induced disease 2019. Br. J. Pharmacol..

[2] Wu Z., McGoogan J.M. (2020). Characteristics of and important lessons from the coronavirus disease 2019 (COVID-19) outbreak in China: Summary of a report of 72,314 cases from the Chinese Center for Disease Control and Prevention. JAMA.

[3] Zhang Q., Bastard P., Liu Z., Le Pen J., Moncada-Velez M., Chen J., Ogishi M., Sabli I.K.D., Hodeib S., Korol C. (2020). Inborn errors of type I IFN immunity in patients with life-threatening COVID-19. Science.

[4] Bastard P., Rosen L.B., Zhang Q., Michailidis E., Hoffman H., Zhang Y., Dorgham K., Philippot Q., Rosain J., Béziat V. (2020). Autoantibodies against type I IFNs in patients with life-threatening COVID-19. Science.

[5] Callard F., Perego E. (2021). How and why patients made Long Covid. Soc. Sci. Med..

[6] Davis H.E., Assaf G.F., McCorkell L., Wei H., Low R.J., Re’em Y., Redfield S., Austin J.P., Akrami A. (2021). Characterizing long COVID in an international cohort: 7 months of symptoms and their impact. EClinicalMedicine.

[7] Chang Y.C., Yu C.J., Chang S.C., Galvin J.R., Liu H.M., Hsiao C.H., Kuo P.H., Chen K.Y., Franks T.J., Huang K.M. (2005). Pulmonary sequelae inconvalescent patients after severe acute respiratory syndrome: Evaluation with thin-section CT. Radiology.

[8] Pan F., Ye T., Sun P., Gui S., Liang B., Li L., Zheng D., Wang J., Hesketh R.L., Yang L. (2020). Time Course of Lung Changes at Chest CT during Recovery from Coronavirus Disease 2019 (COVID-19). Radiology.

[9] Polidoro R.B., Hagan R.S., de Santis Santiago R., Schmidt N.W. (2020). Overview: Systemic Inflammatory Response Derived From Lung Injury Caused by SARS-CoV-2 Infection Explains Severe Outcomes in COVID-19. Front. Immunol..

[10] Smilowitz N.R., Kunichoff D., Garshick M., Shah B., Pillinger M., Hochman J.S., Berger J.S. (2021). C-reactive protein and clinical outcomes in patients with COVID-19. Eur. Heart J..

[11] Mehta P., McAuley D.F., Brown M., Sanchez E., Tattersall R.S., Manson J.J. (2020). COVID-19: Consider cytokine storm syndromes and immunosuppression. Lancet.

[12] Jalkanen J., Hollmén M., Jalkanen S. (2020). Interferon beta-1a for COVID-19: Critical importance of the administration route. Crit. Care.

[13] Buttmann M., Merzyn C., Rieckmann P. (2004). Interferon-β induces transient systemic IP-10/CXCL10 chemokine release in patients with multiple sclerosis. J. Neuroimmunol..

[14] Terlizzi M., Molino A., Colarusso C., Donovan C., Imitazione P., Somma P., Aquino R.P., Hansbro P.M., Pinto A., Sorrentino R. (2018). Activation of the Absent in Melanoma 2 Inflammasome in Peripheral Blood Mononuclear Cells From Idiopathic Pulmonary Fibrosis Patients Leads to the Release of Pro-Fibrotic Mediators. Front. Immunol..

[15] Borthwick L.A. (2016). The IL-1 cytokine family and its role in inflammation and fibrosis in the lung. Semin. Immunopathol..

[16] Cavalli G., Colafrancesco S., Emmi G., Imazio M., Lopalco G., Maggio M.C., Sota J., Dinarelloh C.A. (2021). Interleukin 1α: A comprehensive review on the role of IL-1α in the pathogenesis and treatment of autoimmune and inflammatory diseases. Autoimmun. Rev..

[17] Nigdelioglu R., Hamanaka R.B., Meliton A.Y., O’Leary E., Witt L.J., Cho T., Sun K., Bonham C., Wu D., Woods P.S. (2016). Transforming growth factor (TGF)-β promotes de Novo serine synthesis for collagen production. J. Biol. Chem..

[18] Carvalho T., Krammer F., Iwasaki A. (2021). The first 12 months of COVID-19: A timeline of immunological insights. Nat. Rev. Immunol..

[19] Tale S., Ghosh S., Meitei S.P., Kolli M., Garbhapu A.K., Pudi S. (2020). Post-COVID-19 pneumonia pulmonary fibrosis. QJM Int. J. Med..

[20] DiPiazza A.T., Graham B.S., Ruckwardt T.J. (2021). T cell immunity to SARS-CoV-2 following natural infection and vaccination. Biochem. Biophys. Res. Commun.

[21] Chung J.Y., Thone M.N., Kwon J.Y. (2021). COVID-19 vaccines: The status and perspectives in delivery points of view. Adv. Drug Deliv. Rev..

[22] Malmström J., Lindberg H., Lindberg C., Bratt C., Wieslander E., Delander E., Särnstrand B., Burns J.S., Mose-Larsen P., Fey S. (2004). Transforming growth factor-beta 1 specifically induce proteins involved in the myofibroblast contractile apparatus. Mol. Cell. Proteom..

