# Supplementary material for: Post-COVID-19 Patients Who Develop Lung Fibrotic-like Changes Have Lower Circulating Levels of IFN-β but Higher Levels of IL-1α and TGF-β
Source: Biomedicines. 2021 Dec 17;9(12):1931. doi: 10.3390/biomedicines9121931 (PMC8698335; doi:10.3390/biomedicines9121931)
Supplement: Supplementary file 1 [file biomedicines-09-01931-s001.zip › biomedicines-1472695-supplementary.pdf]

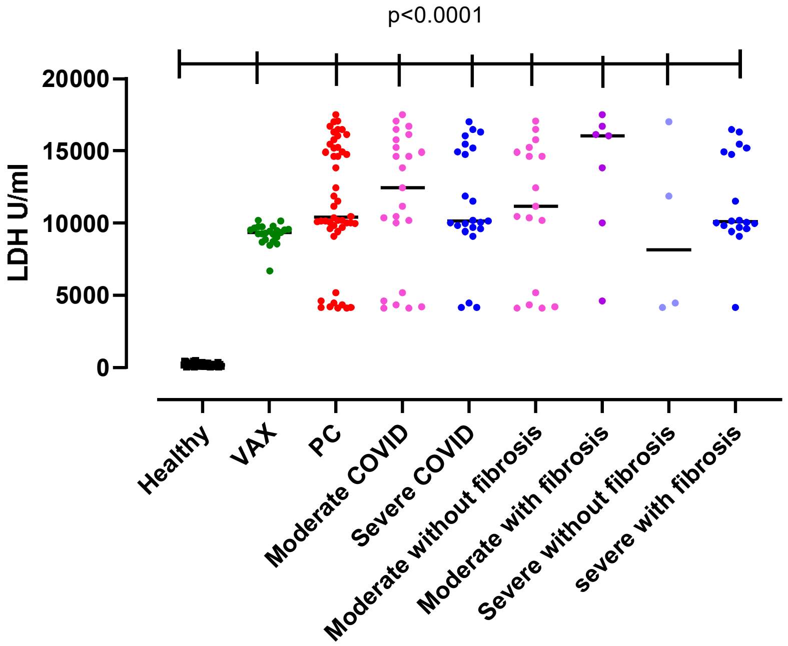

Figure S1. Lactate dehydrogenate (LDH) levels were increased in post COVID-19 (PC) patients. Plasma levels of LDH were measured in healthy and vaccinated (VAX) subjects (black and green dots, respectively) and PC patients (red dots). LDH values were evaluated according to the grade of disease (moderate or severe, pink and blue dots, respectively). Data are expressed as median. Statistical analysis was performed according to the Mann–Whitney U test

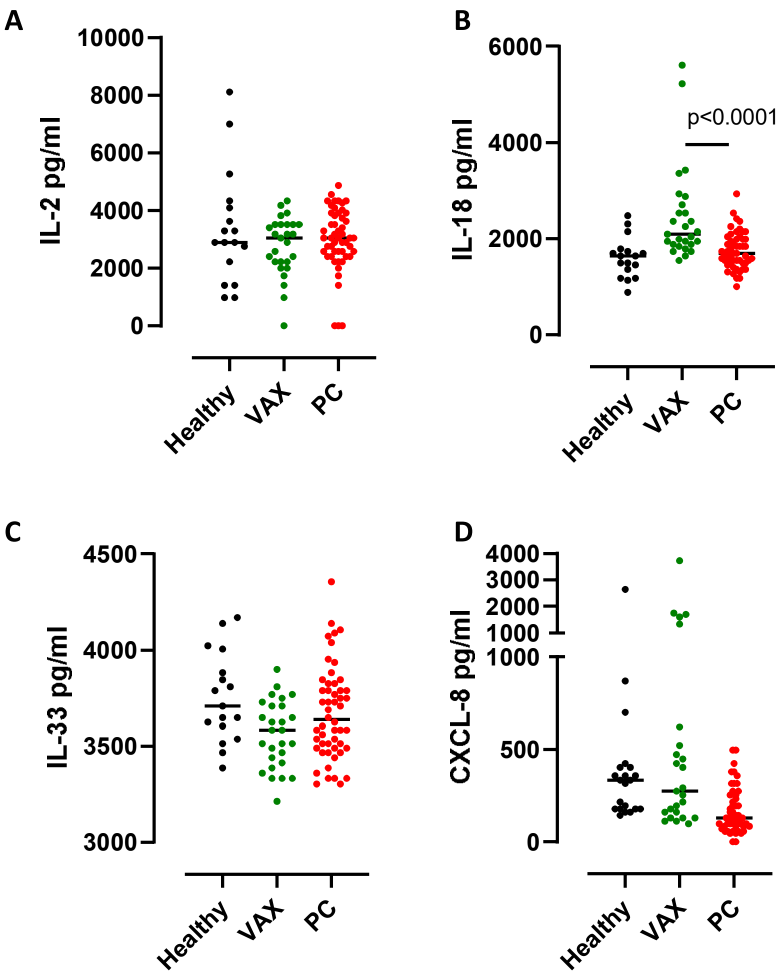

Figure S2. Circulating levels of IL-2, IL-18, IL-33, and CXCL8 in PC patients and in healthy and vaccinated subjects. Plasma levels of IL-2 (A), IL-18 (B), IL-33 (C), and CXCL8 (D) were measured in healthy and vaccinated (VAX) subjects (black and green dots, respectively) and post COVID-19 (PC) patients (red dots)
